# Pupil-mimicry conditions trust in partners: moderation by oxytocin and group membership

**DOI:** 10.1098/rspb.2016.2554

**Published:** 2017-03-15

**Authors:** Mariska E. Kret, Carsten K. W. De Dreu

**Affiliations:** 1 Leiden Institute of Psychology, Leiden University, Leiden, The Netherlands; 2 Leiden Institute for Brain and Cognition (LIBC), Leiden, The Netherlands; 3 Center for Experimental Economics and Political Decision Making, University of Amsterdam, Amsterdam, The Netherlands

**Keywords:** oxytocin, pupil dilation, social decisions, economic game, eye signal, mimicry

## Abstract

Across species, oxytocin, an evolutionarily ancient neuropeptide, facilitates social communication by attuning individuals to conspecifics' social signals, fostering trust and bonding. The eyes have an important signalling function; and humans use their salient and communicative eyes to intentionally and unintentionally send social signals to others, by contracting the muscles around their eyes and pupils. In our earlier research, we observed that interaction partners with dilating pupils are trusted more than partners with constricting pupils. But over and beyond this effect, we found that the pupil sizes of partners synchronize and that when pupils synchronously dilate, trust is further boosted. Critically, this linkage between mimicry and trust was bound to interactions between ingroup members. The current study investigates whether these findings are modulated by oxytocin and sex of participant and partner. Using incentivized trust games with partners from ingroup and outgroup whose pupils dilated, remained static or constricted, this study replicates our earlier findings. It further reveals that (i) male participants withhold trust from partners with constricting pupils and extend trust to partners with dilating pupils, especially when given oxytocin rather than placebo; (ii) female participants trust partners with dilating pupils most, but this effect is blunted under oxytocin; (iii) under oxytocin rather than placebo, pupil dilation mimicry is weaker and pupil constriction mimicry stronger; and (iv) the link between pupil constriction mimicry and distrust observed under placebo disappears under oxytocin. We suggest that pupil-contingent trust is parochial and evolved in social species in and because of group life.

## 1. Introduction

Pivotal to social life is the ability to trust others—to have a positive expectation that others will cooperate and not exploit us [1–3]. Sometimes, assessments of trustworthiness derive from an elaborate evaluation of the risks involved and the extent to which possible benefits outweigh potential losses [4,5]. Often, trust is intuitive, affect-based and reflecting a ‘gut feeling’ based on the partner's physical features [6–10]. Across species, such ‘gut feeling’ may derive from a variety of sources, such as partners' bodily scents (in rodents [11,12]; in humans [13]), posture (in rodents [14]; in humans [15,16,17,18]) and emotional vocalizations (in rodents [19]; in chimpanzees [20]; in humans [21]).

One important yet understudied physical characteristic that may be used to form intuitive assessments of the partner's trustworthiness is the eye. In humans, the eye has a morphology that is unique among primates [22,23]. The eyes are crucially important during social communication and provide information to regulate interaction, express intimacy, exercise social control, and facilitate service and task goals [24]. Not surprisingly, the making of eye-contact provides a powerful mode of establishing each other's emotions and intentions [25], which can influence trust-based decisions [26].

During eye-contact, pupil sizes tend to synchronize across partners so that dilating pupils induce pupil dilation in the partner, and constricting pupils increase pupil constriction in the partner [27]. This pupil mimicry is already present during the first months of life [28] and is an evolutionarily old phenomenon shared with other species [29]. Recently, pupil mimicry in humans has been shown to relate to intuitive assessments of a partner's trustworthiness [26], as Dutch participants played trust-games with partners of whom just the eye region was visible and in which the pupils were manipulated to change in size. Results showed that participants' own pupils dilated synchronously with their partner's pupils. Importantly, this correlated with the extent to which participants trusted their partner, especially when partners also were from Caucasian descent (henceforth ingroup). With partners from Japanese descent (henceforth outgroup), there was no linkage whatsoever between pupil mimicry and trust.

Although pupil mimicry reflects an autonomic response that emerges outside conscious awareness and deliberate control, the mechanisms that link pupil mimicry to trust remain poorly understood [26]. One possibility is that the linkage is conditioned by oxytocin, an evolutionary ancient neuropeptide that acts as hormone and neurotransmitter. This possibility follows from two lines of evidence. First, the making of eye contact fosters the release of oxytocin in humans as well as in other species including dogs [30]. Furthermore, in closely bonded partners such as parents and their offspring, oxytocin levels tend to synchronize. This holds for humans [31], as well as for family groups of cooperatively breeding marmoset monkeys [32]. Second, oxytocin is intimately involved in the regulation of social bonding, affiliation and prosocial behaviour, again across a wide range of mammalian species. For example, oxytocin boosts pair-bond formation and paternal behaviour in prairi voles [33,34], ‘tend-and-defend’ patterns of affiliation in chimpanzees [35,36], and social approach and affiliation with conspecifics in dogs [37]. In humans, oxytocin increases sensitivity to one's partner's emotion expressions [38–40].

While eye-contact between partners promotes the release of oxytocin, and oxytocin levels synchronize during close partner interactions and appear to facilitate pro-social exchange and affiliation, there is evidence also that these effects of oxytocin are ingroup bounded [41]. In both humans and chimpanzees, oxytocin increases trust and cooperation with familiar others and members of one's ingroup [35,41–45]. At the same time, oxytocin appears to upregulate defensive shielding and vigilance vis-à-vis outsiders and unfamiliar others. This tendency has been observed in humans [43], marmosets [46], California mice [47], female rats [48], prairie voles [49] and wild chimpanzees [36]. For example, one study demonstrated that prairie voles show increased partner-directed grooming toward familiar but not unfamiliar conspecifics that experienced an unobserved stressor, but that blocking the oxytonergic circuitry abolished this partner-directed response [49]. Also, Samuni *et al*. [36] showed stronger patterns of oxytocin-mediated ingroup affiliation among wild chimpanzees prior to intergroup fighting.

Taken together, there is reason to assume that the pupil dilation mimicry–trust linkage that emerges with ingroup partners is conditioned by oxytocin. We tested this possibility here, with healthy males and females. We focused on humans because of the catching morphology of the human eye, which sets it apart from most other species [22], and because humans have frequent encounters with unfamiliar others. We included both male and female subjects because animal studies show sex differences in how oxytocin influences behaviour [50,51]. Thus, a more exploratory goal of the present study was to examine possible sex differences in the interplay between pupil mimicry, oxytocin and ingroup trust (see also [52–54]).

## 2. Methods

In two separate sessions, participants received oxytocin or placebo and made investment decisions in incentivized trust games with different virtual ingroup or outgroup partners. Per game or trial, participants could invest €0, €2, €4 or €6 in their partner, knowing that investments would be tripled (i.e. €2 becomes €6 for the trustee), and that by the end of the experiment their earnings would be paid out in the form of a bonus. They did not receive feedback regarding trustees' payback decisions during the experiment. Prior to decision-making, participants viewed a short clip of their partner's eye region in which pupils dilated, constricted or remained static.

### (a) Participants

Fifty-nine students (22 years old; 28 males) without neurological or psychiatric history participated in two 1 h sessions with two weeks in-between. The sample size is comparable with our earlier study on pupil mimicry [26] as well as with studies on oxytocin and human decision-making [43,44,55,56]. In both sessions participants were placed in the role of investor, yet in one session they received oxytocin and in the other placebo (double-blind, randomized cross-over). All participants were born and raised in the Netherlands, with Dutch parents.

### (b) Medication

Before the experiment, participants completed a medical screening, and we invited those without a significant neurological or psychiatric history, who did not use prescription-based medication, smoked less than five cigarettes per day and did not report drug or alcohol abuse. Eligible participants were assigned to a session and instructed to refrain from smoking or drinking (except water) for 2 h before the experiment. At the beginning of each session, participants self-administered a single intranasal dose of 24 IU oxytocin (Syntocinon spray, Novartis; three puffs per nostril, each with 4 IU oxytocin, with 2 min interval between puffs) or placebo. To avoid any subjective effects (for example, olfactory effects) other than those caused by oxytocin, the placebo contained all the active ingredients except for the neuropeptide. Placebos were delivered in the same bottles as Syntocinon. Thus, participants and experimenters were ignorant about treatment conditions [42].

### (c) Stimuli

**Figure 1.**
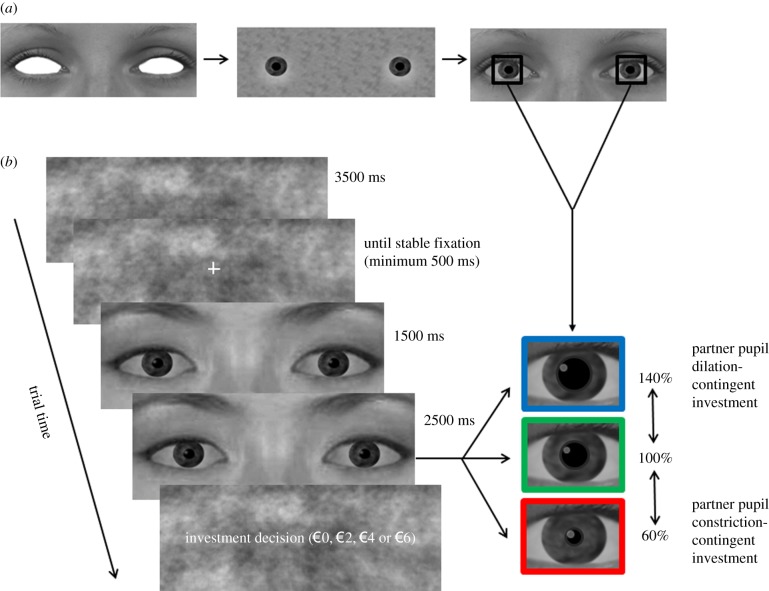
(*a*) Stimulus characteristics and (*b*) sample trial sequence. To create partner stimuli, we removed the eyes from pictures of the eye regions of faces and then added the same eye white, iris and pupil to each stimulus (independent of partner's group). In each trial, a scrambled image of a stimulus was presented for 4000 ms. The scrambled image was then replaced by the stimulus itself. In all conditions, the stimulus remained static for the first 1500 ms, but in the dilation and constriction conditions, the pupils gradually changed in size over the following 1500 ms and then remained at that size during the final 1000 ms (in the static condition, pupils remained at the same size throughout the trial). Finally, a screen appeared asking participants to decide to transfer €0, €2, €4 or €6 to their partner. (Online version in colour.)

The stimulus material was similar to that used in our previous study [26]. Pictures of the eye region of Dutch (ingroup) and Japanese (outgroup) actors were included. Within the eye region, an artificial pupil was added to dynamically change in size. Specifically, after static presentation for 1500 ms, the pupil remained either static or dilated or constricted within the physiological range over 1500 ms. In the last second, the pupils were static. The eye images appeared life-size on the computer screen. We verified that images of the partners reflected ingroup/outgroup differences. (figure 1; electronic supplementary materials).

### (d) Procedure

Participants were seated individually and provided written informed consent. The experimenter then handed participants a nasal spray and left after they self-administered oxytocin or placebo. Because treatment effects tend to emerge especially after 30 min of loading time [57], participants continued with otherwise irrelevant questionnaires and survey. After 30 min elapsed, the actual experiment began. The participant sat at a distance of 60 cm from the computer screen and read instructions. They read that they had €6, of which they could invest €0, €2, €4 or €6 in partners. Each investment would be tripled. It was emphasized that the partners had participated earlier and indicated for each possible investment how much they would reciprocate (this was indeed the case, with additional participants acting as trustees in an earlier session, and participants' investment decisions were coupled to these partner decisions to calculate actual payoffs). Participants were further told that recordings had been made of these partners, that these recordings had been manipulated and that they would see them before they had to make their investment decision, to give them an idea about what sort of person they would interact with. They were further told that Dutch and Japanese students were participating and were asked to indicate via button-press whether they themselves were from the University of Amsterdam or from the University of Tokyo.

After participants had correctly answered three practice questions, they started with a nine-point calibration of the eye-tracker (EyeLink, SR Research, Ottawa, Canada; screen-type: ViewSonic VG732M, 1280 × 1024 pixels), followed by the start of the first trial. To minimize pupil constriction following new information that is presented on the screen, a trial started with the presentation of a unique Fourier-scrambled image of the actual stimulus. This scrambled image was presented for 3500 ms. After 3500 ms, a fixation cross appeared on top of the scrambled image and lasted until participants fixated properly. Next, an image of partners' eyes with dilating, static or constricting pupil size was presented for 4000 ms. After the offset of the image, participants were instructed to make an investment decision.

After the experiment, participants were asked whether they had noted anything special about the partners' eyes and what they thought the study was about. Although participants indicated being aware of the dynamics in partners' pupil size, none of them suspected that we were interested in pupil mimicry and its link with trust.

### (e) Trustee decisions

Participant (investors) payments were based on back-transfer decisions (i.e. decisions about the amount they would transfer back to their partners) made by 15 other students (2 males, 13 females, mean age 24 years, range 18–55 years old) in the role of trustee, who were given a form with 10 investment decisions of others (€0–€10) and asked how much they would reciprocate given a certain investment. These back-transfer decisions were randomly chosen and paired to those made by participants in the main experiment, to calculate actual earnings. For each trial, we randomly drew a decision to calculate participants' earnings after the experiment was over.

### (f) Statistical analyses

Data were analysed with linear mixed multi-level models, allowing the estimation of individual differences by modelling variances of slopes and intercepts. Model selection started with a full model including as fixed factors the partner's group (ingroup/outgroup), partner's pupil (dilating/static/constricting), participant's treatment (oxytocin/placebo) and their interactions. After each single removal of one factor, we compared the more parsimonious model with the more complex model with a log-likelihood ratio test (LLRT). If the result of the LLRT determined that the term under consideration did not increase model fit, it was removed; otherwise it was kept. After defining the fixed factors, model selection proceeded with the random factors. We first added four random factors (a random slope and intercept for each subject and for each subject × trial) and similar to the back-fitting process of the fixed factors, defined the random factors. Crucially, we were able to include time as a repeated factor with a first-order autoregressive (AR1) covariance structure to control for auto-correlation with regard to time in the pupillometry analyses. These models additionally included linear, quadratic and cubic polynomials as fixed and random factors to precisely model the slope of participants pupil size. Given the large number of factors, we focus on effects that include the factor partner pupil. Especially the pupillometry models contain a large number of fixed factors due to the interactions with the polynomials. For that reason, when modelling participants' pupil size, we additionally limit ourselves to effects that survive a threshold of *p* < 0.005 (full reports are provided in the electronic supplementary material, Results).

Pupil responses were analysed over the last 2500 ms of stimulus presentation (i.e. from the moment partners' pupils started to change in size). Pupil-size data were down-sampled to 100 ms timeslots and smoothed with a 10th-order low-pass Butterworth filter. The 500 ms just before the partners' pupils started to change were used as baseline and subtracted from subsequent data points.

## 3. Results

### (a) Investments

**Figure 2.**
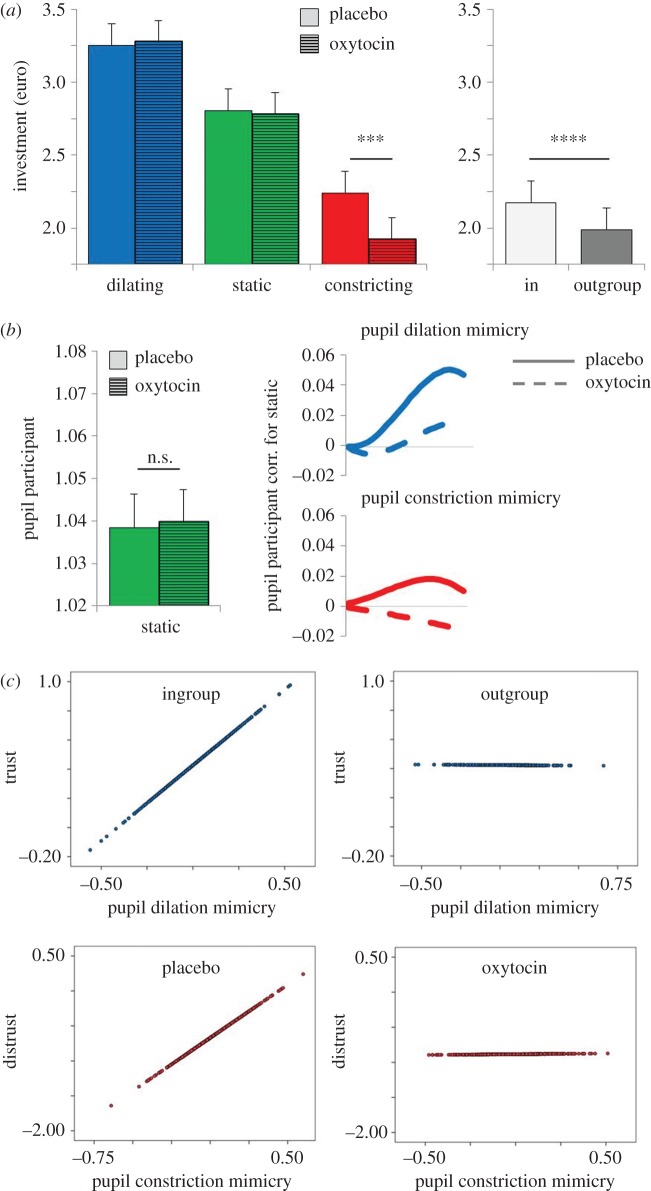
(*a*–*c*) All visualizations represent predicted data by the best-fitting statistical models. Error bars signify the standard error of the means. Dilation (constriction) mimicry (*b*,*c*) was measured by subtracting participants' pupil size when partners' pupils were static from their pupil size when partner's pupils dilated (constricted). Trust (distrust) in (*c*) denotes participants' investments in partners with dilating (constricting) pupils minus investments in partners with static pupils. *p < 0.05; **p < 0.01. (Online version in colour.)

Effects of partner pupil (*F*_2,5.213_ = 247,184, *p* < 0.001) and group (*F*_1,5.213_ = 18,332, *p* < 0.001) showed that partners with dilating pupils and partners from the ingroup were trusted more than partners with static or constricting pupils and partners from the outgroup. The significant treatment × partner pupil interaction (*F*_2,5.213_ = 6.683, *p* = 0.001) showed that participants given oxytocin invested less in partners with constricting pupils than participants given placebo (figure 2*a*). This effect was further qualified by a treatment × sex participant × pupil partner interaction. The effect shown in figure 2*a* was present for male, but not for female participants (males *p* = 0.005; females *p* = 0.276). In addition, whereas males given oxytocin rather than placebo trusted partners with dilating pupils (*p* = 0.010), females trusted partners with dilating pupils less under oxytocin than under placebo (*p* = 0.031). There were no effects of treatment on investments when partner's pupils remained static (all *p*s > 0.602; electronic supplementary material, figure S1 and table S1).

### (b) Pupil mimicry

To examine whether the current experiment replicates the results reported in [26], we computed their analytic model (i.e. first without the factors treatment and sex). As in that study, we find evidence for pupil mimicry, as is demonstrated by a main effect of partner pupil (*F*_2,5630.400_ = 9.731, *p* < 0.001) and a two-way interaction between pupil partner and the linear term (*F*_2,82975.646_ = 75.904, *p* < 0.001). Thus, participants' pupils were larger and dilated faster when observing a partner with dilating as compared with static or constricting pupils (electronic supplementary material, table S2). These effects were independent of looking times (electronic supplementary material, tables S8 and S9) or potential differences between the sexes or treatment groups in their level of tonic arousal (electronic supplementary material, table S10). With this successful replication of earlier findings, we proceeded with testing the moderating influence of oxytocin, and explored effects of partner and participant sex. Results are described separately for pupil dilation mimicry and for pupil constriction mimicry.

### (c) Pupil dilation mimicry

A main effect of pupil partner showed that participants' pupils were larger when viewing partners with dilating as compared to static pupils (*F*_1,3736.291_ = 16.263, *p* < 0.001). A pupil partner × linear term interaction showed that participants' pupils also increased faster over stimulus presentation time than when viewing static pupils (*F*_1,56039.191_ = 111.880, *p* < 0.0001). A treatment × pupil partner interaction, in conjunction with the significant treatment × pupil partner × linear term interaction (*F*_1,50754.286_ = 16.839, *p* < 0.001 and *F*_1,3736.808_ = 8.877, *p* = 0.003) showed that pupil dilation mimicry was weaker under oxytocin as compared with placebo (figure 2*b*; electronic supplementary material, table S3). Effects of sex of partner and participant on pupil mimicry did not survive our statistical threshold and are reported in electronic supplementary material, table S3.

In a separate linear mixed multi-level model, we investigated the effects of pupil dilation mimicry, partner group, treatment and their interactions (fixed factors) on investment decisions (dependent variable). As in [26], we find that pupil dilation mimicry increased partner–pupil contingent trust in interactions with ingroup partners (*F*_1,718.900_ = 4.367, *p* = 0.037), but not in interactions with outgroup partners (*F*_1,710.885_ = 0.000, *p* = 0.989). Treatment did not modulate this general tendency, and nor did sex of partner or sex of participant (electronic supplementary material, tables S4 and S5).

### (d) Pupil constriction mimicry

A pupil partner × quadratic term interaction showed that when viewing partners with constricting pupils, participants' pupils initially increased in size and then quickly decreased, resulting in a greater curvature of the slope as compared with when viewing partners with static pupils (*F*_1,15.381_ = 225966.099, *p* < 0.001). The treatment × pupil partner × linear term interaction, in conjunction with the treatment × pupil partner × quadratic term interaction, showed that pupil constriction mimicry was stronger under oxytocin as compared with placebo (*F*_1,15.975_ = 22590.950, *p* < 0.001; *F*_1,9.794_ = 225963.865, *p* = 0.002; figure 2*b*).

As noted, our design enabled us to explore effects of sex of partner and participant on pupil mimicry. We observed, first of all, a sex participant × sex partner × pupil partner interaction, showing that pupil constriction mimicry was stronger during interactions with a partner of the opposite as compared to the same sex (*F*_1,9.040_ = 225748.689, *p* = 0.003). Second, there was a sex participant × pupil partner × group partner × quadratic term interaction (*F*_1,8.981_ = 225972.102, *p* = 0.003). This effect was mainly driven by male participants whose pupils, after an initial increase in size, started to constrict following outgroup eyes with static pupils (electronic supplementary material, table S6).

In a separate model, we investigated the putative relationship between pupil constriction mimicry, partner group, treatment (fixed factors) and investments (dependent variable). Results showed an interaction between treatment and pupil constriction mimicry (*F*_1,1441.258_ = 7.053, *p* = 0.008), demonstrating that among participants given placebo, more constriction mimicry associated with lower trust (*F*_1,716.611_ = 8.445, *p* = 0.004); under oxytocin, this linkage disappeared (*F*_1,735.116_ = 0.002, *p* = 0.965; figure 2*c*; electronic supplementary material, table S7).

A summary of the key findings is provided in table 1.

**Table 1.** Summary of results. Overview of the main results of the study. dil., dilating; con, constricting; n.s., not significant.

fixed factors	investments/trust	dil. mimicry	con. mimicry
partner pupil	dil. > static > con.	dil. > static	n.s.
treatment	oxytocin = placebo	n.s.	n.s.
partner pupil × partner group	n.s.	n.s.	n.s.
partner pupil × treatment	oxytocin lowers trust in con. pupils	dil. mimicry: oxytocin < placebo	con. mimicry: oxytocin > placebo
partner pupil × partner sex	n.s.	n.s.	n.s.
partner pupil × participant sex	n.s.	n.s.	n.s.
treatment × pupil partner × sex participant	oxytocin boosts trust in dil. pupils in males, but lowers it in females	n.s.	n.s.
sex participant × sex partner × pupil partner	n.s.	n.s.	con. mimicry: opposite > same sex
pupil dil. mimicry—trust linkage	pupil dil. mimicry predicts trust in ingroup		
pupil con. mimicry—distrust linkage	pupil con. mimicry predicts distrust under placebo		

## 4. Discussion

Across species, oxytocin can promote a wide range of affiliative behaviours including cooperation and trust [35,37,46], but depending on the context, can also have antisocial effects and reduce cooperation and trust [36,45,58,59]. The current study confirmed that quick and intuitive decisions to trust are influenced by (i) group membership of the trustee, (ii) trustee's pupil size and (iii) participants' tendency to mimic changes in trustees pupil size, and (iv) that both oxytocin and sex of participant and trustee further moderated these effects. We show that pupil size plays an important role during social interactions. Below we argue that pupil size may be a physiological marker of trust in other social species than humans as well.

On a daily basis, social animals decide quickly and intuitively whether or not to trust an interaction partner. Especially in humans, this is an important ability given that most people live in large cities where they know only a very small percentage of those they encounter in daily life. Yet although the way humans live today is unique compared with other animals, where unfamiliar individuals often pose genuine threats to the sorts of small, tightly bound groups of intimates, it is important to note that the human genome developed within much smaller, closely bound groups of people where encounters with strangers were more rare [60]. It stands to reason that both the neurocognitive mechanisms and the types of conspecifics' signals humans use when making trust decisions regarding strangers are shared with other social species.

In the instance of being confronted with a stranger, we mostly rely on the physical characteristics of the other. In humans and non-human primates one heuristic for whether to trust another individual is to categorize him or her as ingroup or outgroup, labels which often predict the tone of a social interaction and the behaviours employed [61,62]. Apart from physical characteristics pointing to familiarity and group membership, other characteristics trust decisions can be based upon are expressions of emotions, social intentions and interest. In that respect the eye region is most expressive and attracts most attention [22,23]. We use the eyes to quickly identify who is who [63], and who belongs to what group [64]; and although humans have particularly expressive eyes, this tendency is not limited to humans. For example, dogs also recognize conspecifics and humans by paying special attention to the eyes [65]. Apart from identity recognition, the eye region is crucially important during social interactions and owes its expressiveness to the fine muscles around the eyes and to the pupils, both expressing internal states of mind including emotions, social interest and trust [66]. Our recent research suggests that these positive signals are partly derived *through* pupil mimicry. That is, by looking into another's dilated pupils, our own pupils dilate in response, providing some sort of feedback signal that presumably helps us to trust the other person better [26]. In a study comparing humans and chimpanzees, Kret *et al.* [29] demonstrated that chimpanzees mimicked the pupil sizes of chimpanzees but not humans, and that humans mimicked the pupil sizes of humans but not chimpanzees. Thus, pupil mimicry, like other forms of mimicry [67], is not uniquely human but is present in at least one other species as well. Because pupil size can provide very useful information about the cognitive or emotional state of the expressor, it is likely to be a physiological marker of trust in a broader range of social species.

The current paper investigated the social and neurobiological boundaries of the relationship between pupil mimicry and trust in humans. Data replicated the effects reported in [26]. As observed earlier, participants trusted partners with dilating pupils more than partners with static or constricting pupils, and trusted partners with constricting pupils less than partners with static pupils (figure 2*a*). Moreover, participants' pupils mimicked the pupil size of their partner (figure 2*b*). Finally, pupil dilation mimicry correlated with higher investments in partners with dilating as compared with static pupils, and this effect was bound to interactions with the ingroup (figure 2*c*).

Data extend this earlier work in three ways. First, oxytocin led male but not female participants to withhold trust from partners with constricting pupils (figure 2*a*). This fits with recent accounts that in case of untrustworthy or unreliable partners, oxytocin can dampen trust [68]. Nature has never rewarded naivety and from an evolutionary perspective it can be inferred that oxytocin does not boost trust unconditionally, but rather that it increases vigilance and a stimulus-congruent ‘sharpening’ of perceived social signals (in humans [46,69,70]; in different species of rodents [46–49]). Along similar lines, Lambert *et al.* [71] observed that oxytocin improved humans' discriminatory ability of untrustworthy but not trustworthy faces. Previous studies in humans investigating the somewhat disputed link between oxytocin and trust mostly included male participants (i.e. [72]). In line with that earlier work, we here find that under oxytocin, male participants trusted partners with dilating pupils more than under placebo. However, as often observed in animal studies [50,51], an opposite pattern was observed for females who trusted these partners less under oxytocin than under placebo (although still more than partners with static or constricting pupils). Similarly, in humans Feng *et al.* [54] showed that oxytocin increased the salience of positive social cues in men, while decreasing their reward value in women (see also [73]).

A second key finding of the current study is that oxytocin weakened pupil dilation mimicry, and strengthened pupil constriction mimicry (figure 2*b*). This finding is similar to a recent study on facial mimicry where oxytocin increased the mimicry of angry faces but had no effect on the mimicry of happy faces [74]. Another recent study showed that oxytocin enhanced inter-brain synchrony during a social coordination but not a control task. This effect, however, was bound to male subjects and was not observed in females as they already had high baseline levels of synchrony [75]. Somewhat relatedly, a recent magneto-encefalogram (MEG) study showed that oxytocin modulated social brain processes differently in combat veterans than in controls when watching images probing social synchrony (in the case of this particular study, coordinated combat action [76]). Thus, oxytocin's effect on mimicry or synchronization may be valence- and saliency-dependent. Research on the effect of oxytocin on synchronous behaviour in other animals is scarce. One study in pigs found no effects of oxytocin on the mimicry of positive or negative emotions, yet some effects were found on the non-treated observing pigs [77].

Finally, whereas pupil dilation mimicry and ingroup partner–pupil contingent trust were not conditioned by oxytocin, oxytocin did condition pupil constriction mimicry and its link with distrust. Specifically, the link between constriction mimicry and lower levels of trust was present under placebo, and absent under oxytocin. Possibly, under oxytocin trust was already so low in interactions with partners with constricting pupils that constriction mimicry could not add much to it.

The current study included only humans, and although we presume similar processes are at stake in other social animals as well, comparative research is needed to confirm this presumption [78]. In a recent study, Engelmann *et al.* [79] tested chimpanzees on a trust game. The results demonstrate that in interacting with a conspecific, chimpanzees showed spontaneous trust in a novel context, flexibly adjusted their level of trust to the trustworthiness of their partner and developed patterns of trusting reciprocity over time. At least in some contexts, then, trust in reciprocity is not unique to humans, but rather has its evolutionary roots in the social interactions of humans' closest primate relatives. As trust and cooperation among human strangers is common [80,81] and evolutionarily advantageous [82], an important question for future studies is whether chimpanzees will trust unfamiliar conspecifics in a trust game and, if so, which cues they rely on when deciding to do so. In fact, our other closest living relatives, bonobos, show striking tolerance towards strangers and share food with them [83,84]. However, we lack experimental evidence regarding how trust develops and whether relationship formation differs between related species with diverse social backgrounds.

Another topic of interest for future studies is the usage of pupillary signals across species. In all mammals, pupillary responses are involuntary, and apart from responding automatically to light levels, also reflect cognitive emotional states (e.g. in macaques [85,86]). But the avian eye is different in this regard, as pupillary size is under *voluntary* control. Rapid fluctuations in pupil size are used in communication, and depending on the context, they indicate positive or negative excitement [87]. How and whether these pupillary signals are picked up by conspecifics and are used in social decisions is not known.

In summary, this study investigated the relationship between pupil mimicry and trust, and its socio-endocrine boundaries in humans. Oxytocin lowered trust extended to partners with constricting pupils and also enhanced the mimicry of this signal. Although oxytocin dampened pupil dilation mimicry, this had no effect on the level of trust that was extended to partners with dilating pupils. Whereas pupil dilation mimicry boosted trust in ingroup members, pupil constriction mimicry related to distrust in ingroup and outgroup partners alike, but only in the placebo condition. The results of the current study underline the importance of the eyes and their subtle and autonomic expressions, reflecting one's own and mirroring others' state of mind during social interactions. Moreover, this study demonstrates that group membership matters even at very basic levels of interaction, and that oxytocin treatment can profoundly change this fundamental relationship between pupil mimicry, on the one hand, and the emergence of interpersonal trust on the other. Pupil mimicry may be a particularly relevant tool for humans to use when trusting strangers, as interactions with strangers occur so frequently. Nonetheless, the mechanism itself may be phylogenetically older and shared across species.

## Supplementary Material

Pupil Synchronization_Oxytocin Trust_SI_2017_01_25.docx

## Data accessibility

Supporting data are available from https://dataverse.harvard.edu/dataset.xhtml?persistentId=doi:10.7910/DVN/ZFXQI6 [88].
