# Supplementary material for: Pupil-mimicry conditions trust in partners: moderation by oxytocin and group membership
Source: Proc Biol Sci. 2017 Mar 15;284(1850):20162554. doi: 10.1098/rspb.2016.2554 (PMC5360920; doi:10.1098/rspb.2016.2554)
Supplement: Pupil Synchronization_Oxytocin Trust_SI_2017_01_25.docx [file rspb20162554supp1.docx]

**Supplementing Methods**

**Stimuli**

Pictures of eight Dutch (ingroup) and eight Japanese (outgroup) actors with neutral expressions were selected from the Amsterdam Dynamic Facial Expression Set (ADFES) and from the JACFE (Matsumoto, 1989; van der Schalk, Hawk, Fischer, & Doosje, 2011). The photos were turned to greyscale, cropped to reveal only the eye region and adjusted for luminance. The eye-white, iris and pupil were erased and next, the average luminance was calculated and each picture was adjusted to the mean. The eyes were then filled with new eye-white and irises (one iris pair from one photo was cut out and used for all). Thus, the same eye-template was used to lay behind the outer edges of the eyes of all stimuli, ingroup and outgroup alike. After these adjustments in Adobe Photoshop, the images were imported as separate layers in Adobe After Effects. In this program, an artificial pupil was added that changed in size within a natural range over a stimulus presentation time of 4s. More precisely, after static presentation for 1,500ms, the pupil remained either static (5mm) or dilated or constricted within the physiological range of 3-7mm for 1,500ms. In the last second, the pupils were static. The change in pupil size did not start immediately, to create the impression in the participant that the change happened in response to the interaction with the participant. A slightly trembling corneal reflection was added to give the stimuli a natural appearance. The pupil dilation or constriction was linear, but the edges were rounded off with an exponential function (natural formula implemented in After Effects). The eye images appeared life-size on the computer screen.

**Validation of ingroup/outgroup categorization**

We verified that images of the partners reflected ingroup/outgroup differences. Students (N = 29; 7 male, 22 female) not involved in the main study evaluated the images in terms of self-other inclusion (Aron, Aron, & Smollan, 1992). Participants rated partners of Western European descent as closer to themselves and to their ingroup than Asian partners, F(1, 497) = 1.846, *p* = .001; F(1, 497) = 8.510, *p* = .004 (Kret et al., 2015).

**Supplementing Results**

**Investment Decisions**

**Figure S1.**


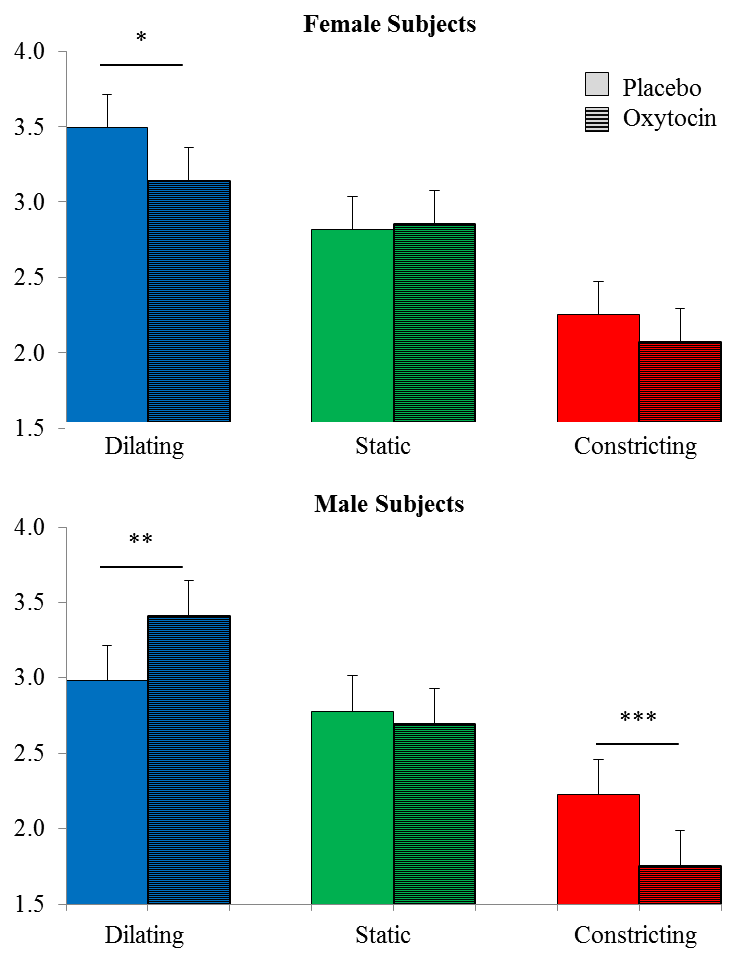


**Investment / Trust**

This model tests effects of the pupil of the partner (dilating, static, constricting), the group of the partner (ingroup, outgroup), the sex of the partner and the sex of the participant and finally effects of treatment (oxytocin, placebo) on investment decisions in the trust game. In bold, effects *p* < .005. We focus on effects that include Pupil Partner as this is of most interest for the current paper and on effects that survive *p* < .005.

**Table S1**

| **Fixed Factors*** | **F** | **df1** | **df2** | ***p*-value** |
| --- | --- | --- | --- | --- |
| Corrected Model | 43,978 | 15 | 5.213 | **.000** |
| Treatment | 1,100 | 1 | 5.213 | .294 |
| Sex Partner | 90,429 | 1 | 5.213 | **.000** |
| Pupil Partner | 247,184 | 2 | 5.213 | **.000** |
| Group Partner | 18,332 | 1 | 5.213 | **.000** |
| Sex Participant | 0,199 | 1 | 5.213 | .656 |
| Treatment * Pupil Partner | 6,683 | 2 | 5.213 | **.001** |
| Treatment * Sex Participant | 0,372 | 1 | 5.213 | .542 |
| Sex Partner * Group Partner | 8,183 | 1 | 5.213 | **.004** |
| Group Partner * Sex Participant | 4,853 | 1 | 5.213 | .028 |
| Treatment * Pupil Partner * Sex Participant | 7,481 | 4 | 5.213 | **.000** |
|  |  |  |  |  |
| **Random Factors** | **Estimate** | **SE** | **Z** | ***p*-value** |
| Variance | 2.491 | .049 | 5.537 | **.000** |
| Var(Intercept) ID | 1.066 | .232 | 4.590 | **.000** |
| Var(Intercept) ID * Session | .210 | .052 | 4.064 | **.000** |

**Pupil Mimicry**

With this model we show we replicated our previous pupil mimicry findings (Kret, Fischer & de Dreu, 2015). The main effect of Pupil Partner, the interaction between Pupil Partner * lin and Pupil Partner * quad show that participant’s pupil size was larger, dilated faster, and showed a more pronounced peak when observing partners pupils which dilated as compared to those which remained static or constricted. This model is followed up by separate models for pupil dilation (dilating and static, Table S3) and constriction mimicry (static and constricting, Table S4).

**Table S2**

| **Fixed Factors** | | | | **F** | | **Df1** | **Df2** | ***p*-value** |
| --- | --- | --- | --- | --- | --- | --- | --- | --- |
| Intercept | | | | 48420.465 | | 1 | 55.412 | **.000** |
| Pupil Partner | | | | 9.731 | | 2 | 5630.400 | **.000** |
| Group Partner | | | | 4.745 | | 1 | 5629.908 | .029 |
| lin | | | | 44.060 | | 1 | 55.087 | **.000** |
| quadr | | | | 35.667 | | 1 | 58.341 | **.000** |
| cub | | | | 1.300 | | 1 | 56.604 | .259 |
| Pupil Partner * Group Partner | | | | .448 | | 2 | 5630.165 | .639 |
| Pupil Partner * lin | | | | 75.904 | | 2 | 82975.646 | **.000** |
| Group Partner * lin | | | | 14.704 | | 1 | 75026.136 | **.000** |
| Pupil Partner * quadr | | | | 5.013 | | 2 | 122456.230 | .007 |
| Group Partner * quadr | | | | .021 | | 1 | 122446.340 | .884 |
| Pupil Partner * Group Partner * quadr | | | | 2.401 | | 2 | 122451.356 | .091 |
| Pupil Partner * cub | | | | 25.858 | | 1 | 118556.952 | **.000** |
|  |  |  |  | |  | ***p*-value** | **95% Confidence Interval** | |
| **Random Factors** |  | **Estimate** | **SE** | | **Z** |  | **Lower** | **Upper Bound** |
| Repeated Measures | AR1 diagonal | .01331 | .0002 | | 62.543 | **.000** | .0129 | .0014 |
|  | AR1 rho | .9766 | .0004 | | 2559.391 | **.000** | .9758 | .9977 |
| Intercept [subject = ID] | Variance | .0009 | .0003 | | 3.368 | **.001** | .0005 | .0016 |
| lin [subject = ID] | Variance | .0054 | .0016 | | 3.297 | **.001** | .0029 | .0098 |
| quadr [subject = ID] | Variance | .0003 | .0001 | | 2.614 | .009 | .0001 | .0006 |
| cub [subject = ID] | Variance | .0002 | <.0001 | | 3.644 | **.000** | .0001 | .0003 |

**Pupil Dilation Mimicry**

There were no interactions between Treatment, Pupil Partner and either Sex Participant and/or Sex Partner that survived the threshold.

**Table S3**

| **Fixed Factors** | | | | **F** | | **Df1** | **Df2** | ***p*-value** |
| --- | --- | --- | --- | --- | --- | --- | --- | --- |
| Intercept | | | | 4569.733 | | 1 | 55.297 | **.000** |
| Sex Participant | | | | .643 | | 1 | 55.296 | .426 |
| Treatment | | | | 5.981 | | 1 | 3786.651 | .015 |
| Sex Partner | | | | 1.981 | | 1 | 3736.963 | .159 |
| Pupil Partner | | | | 16.263 | | 1 | 3736.291 | **.000** |
| Group Partner | | | | 4.930 | | 1 | 3735.991 | .026 |
| lin | | | | 55.740 | | 1 | 55.675 | **.000** |
| quadr | | | | 28.071 | | 1 | 56.575 | **.000** |
| cub | | | | 2.809 | | 1 | 55.738 | .099 |
| Sex Participant * Treatment | | | | 4.497 | | 1 | 3786.647 | .034 |
| Sex Participant * Sex Partner | | | | 1.426 | | 1 | 3736.951 | .233 |
| Sex Participant * Pupil Partner | | | | .003 | | 1 | 3736.600 | .955 |
| Sex Participant * Group Partner | | | | .156 | | 1 | 3735.987 | .693 |
| Sex Participant * lin | | | | .121 | | 1 | 55.674 | .729 |
| Treatment * Sex Partner | | | | .153 | | 1 | 3736.889 | .696 |
| Treatment * Pupil Partner | | | | 8.877 | | 1 | 3736.808 | **.003** |
| Treatment * Group Partner | | | | .269 | | 1 | 3478.804 | .604 |
| Treatment * lin | | | | 6.719 | | 1 | 5265.173 | .010 |
| Sex Partner * Pupil Partner | | | | .245 | | 1 | 3479.195 | .621 |
| Sex Partner * Group Partner | | | | 2.808 | | 1 | 3736.766 | .094 |
| Sex Partner * lin | | | | 1.148 | | 1 | 56052.521 | .284 |
| Pupil Partner * Group Partner | | | | .134 | | 1 | 3736.627 | .714 |
| Pupil Partner * lin | | | | 111.880 | | 1 | 56039.191 | **.000** |
| Group Partner * lin | | | | 11.245 | | 1 | 5605.380 | **.001** |
| Sex Participant * Treatment * Sex Partner | | | | 1.211 | | 1 | 3736.892 | .271 |
| Sex Participant * Treatment * Pupil Partner | | | | .658 | | 1 | 3478.989 | .417 |
| Sex Participant * Treatment * Group Partner | | | | .089 | | 1 | 3478.800 | .766 |
| Sex Participant * Treatment * lin | | | | 3.520 | | 1 | 52649.640 | .061 |
| Sex Participant * Sex Partner * Pupil Partner | | | | 1.897 | | 1 | 3479.185 | .169 |
| Sex Participant * Sex Partner * lin | | | | .219 | | 1 | 56052.062 | .640 |
| Sex Participant * Pupil Partner * Group Partner | | | | .436 | | 1 | 3736.633 | .509 |
| Sex Participant * Pupil Partner * lin | | | | .943 | | 1 | 56039.118 | .332 |
| Treatment * Sex Partner * Pupil Partner | | | | .714 | | 1 | 3478.978 | .398 |
| Treatment * Sex Partner * Group Partner | | | | .193 | | 1 | 3478.938 | .661 |
| Treatment * Sex Partner * lin | | | | .035 | | 1 | 56051.920 | .851 |
| Treatment * Pupil Partner * lin | | | | 16.839 | | 1 | 50754.286 | **.000** |
| Sex Partner * Pupil Partner * lin | | | | .888 | | 1 | 5605.203 | .346 |
| Sex Participant * Treatment * Pupil Partner * lin | | | | 3.901 | | 1 | 50753.887 | .048 |
| Sex Participant * Sex Partner * Pupil Partner * lin | | | | 7.389 | | 1 | 56044.330 | .007 |
| Treatment * Sex Partner * Pupil Partner * lin | | | | 4.171 | | 1 | 50754.161 | .041 |
| Sex Participant * quadr | | | | 2.680 | | 1 | 56.575 | .107 |
| Treatment * quadr | | | | .000 | | 1 | 42511.982 | .985 |
| Sex Partner * quadr | | | | .775 | | 1 | 81543.041 | .379 |
| Pupil Partner * quadr | | | | .029 | | 1 | 81537.588 | .865 |
| Group Partner * quadr | | | | 1.063 | | 1 | 81534.262 | .302 |
| Sex Participant * Treatment * quadr | | | | .588 | | 1 | 42514.855 | .443 |
| Sex Participant * Sex Partner * quadr | | | | .012 | | 1 | 81542.946 | .913 |
| Sex Participant * Pupil Partner * quadr | | | | 1.135 | | 1 | 81537.076 | .287 |
| Sex Participant * Group Partner * quadr | | | | .044 | | 1 | 81534.287 | .834 |
| Treatment * Sex Partner * quadr | | | | .493 | | 1 | 81541.288 | .483 |
| Treatment * Pupil Partner * quadr | | | | 6.271 | | 1 | 8154.586 | .012 |
| Sex Partner * Group Partner * quadr | | | | 4.130 | | 1 | 81543.399 | .042 |
| Pupil Partner * Group Partner * quadr | | | | .537 | | 1 | 81534.052 | .464 |
| Sex Participant * Treatment * Sex Partner * quadr | | | | 4.027 | | 1 | 81541.283 | .045 |
| Sex Participant * Pupil Partner * Group Partner * quadr | | | | 4.415 | | 1 | 81534.062 | .036 |
| Sex Participant * cub | | | | .026 | | 1 | 55.738 | .872 |
| Treatment * cub | | | | 1.484 | | 1 | 4096.671 | .223 |
| Sex Partner * cub | | | | .208 | | 1 | 78957.071 | .649 |
| Pupil Partner * cub | | | | 46.558 | | 1 | 7895.415 | **.000** |
| Group Partner * cub | | | | .505 | | 1 | 78953.854 | .477 |
| Sex Participant * Treatment * cub | | | | .275 | | 1 | 40963.189 | .600 |
| Sex Participant * Sex Partner * cub | | | | 3.172 | | 1 | 78955.763 | .075 |
| Sex Participant * Pupil Partner * cub | | | | .728 | | 1 | 7895.140 | .393 |
| Sex Participant * Group Partner * cub | | | | 4.199 | | 1 | 81257.120 | .040 |
| Treatment * Sex Partner * cub | | | | .532 | | 1 | 78953.758 | .466 |
| Sex Partner * Pupil Partner * cub | | | | 6.698 | | 1 | 7896.473 | .010 |
| Sex Participant * Treatment * Group Partner * cub | | | | 9.622 | | 1 | 81257.551 | **.002** |
| Sex Participant * Sex Partner * Pupil Partner * cub | | | | 4.878 | | 1 | 7896.526 | .027 |
| Treatment * Sex Partner * Group Partner * cub | | | | 4.338 | | 2 | 81262.954 | .013 |
|  |  |  |  | |  | ***p*-value** | **95% Confidence Interval** | |
| **Random Factors** |  | **Estimate** | **SE** | | **Z** |  | **Lower** | **Upper Bound** |
| Repeated Measures | AR1 diagonal | .014 | .000 | | 5.795 | **.000** | .013 | .014 |
|  | AR1 rho | .977 | .000 | | 2127.501 | **.000** | .976 | .978 |
| Intercept [subject = ID] | Variance | .001 | .000 | | 4.458 | **.000** | .001 | .002 |
| lin [subject = ID] | Variance | .008 | .002 | | 4.920 | **.000** | .005 | .012 |
| quadr [subject = ID] | Variance | .000 | .000 | | 4.029 | **.000** | .000 | .001 |
| cub [subject = ID] | Variance | .000 | .000 | | 3.963 | **.000** | .000 | .000 |

**Pupil Dilation Mimicry- Pupil-Contingent Trust Linkage**

There were no interactions between Treatment, Pupil Dilation Mimicry and either Sex Participant and/or Sex Partner.

| **Table S4 Pupil Dilation Mimicry- Pupil-Contingent Trust Linkage Ingroup** | | | | | |  |
| --- | --- | --- | --- | --- | --- | --- |
|  |  |  |  |  |  |  |
| **Fixed Factors** | **F** | **df1** | **df2** | ***p*-value** |  |  |
| Intercept | 15.991 | 1 | 99.600 | **.000** |  |  |
| Dilation Mimicry | 4.367 | 1 | 718.900 | .037 |  |  |
|  |  |  |  |  | **95% Confidence Interval** | |
| **Random Factors** | **Estimate** | **SE** | **Z** | ***p*-value** | **Lower** | **Upper Bound** |
| Residual | 3.559 | .197 | 18.109 | **.000** | 3.194 | 3.965 |
| Intercept [subject = ID * Session] Variance | .677 | .165 | 4.100 | **.000** | .420 | 1.092 |
|  |  |  |  |  |  |  |
|  |  |  |  |  |  |  |
|  |  |  |  |  |  |  |
| **Table S5 Pupil Dilation Mimicry- Pupil-Contingent Trust Linkage Outgroup** | | | | | | |
|  |  |  |  |  |  |  |
| **Fixed Factors** | **F** | **df1** | **df2** | ***p*-value** |  |  |
| Intercept | 14.114 | 1 | 97.858 | **.000** |  |  |
| Dilation Mimicry | .032 | 1 | 704.314 | .858 |  |  |
| Treatment | .018 | 1 | 97.626 | .895 |  |  |
| Sex Participant | .210 | 1 | 96.814 | .648 |  |  |
| Treatment * Sex Participant | 4.604 | 1 | 96.870 | .034 |  |  |
|  |  |  |  |  | **95% Confidence Interval** | |
| **Random Factors** | **Estimate** | **SE** | **Z** | ***p*-value** | **Lower** | **Upper Bound** |
| Residual | 3.513 | .195 | 17.977 | **.000** | 3.150 | 3.917 |
| Intercept [subject = ID * Session] Variance | .815 | .186 | 4.378 | **.000** | .521 | 1.276 |

**Pupil Constriction Mimicry**

There were no interactions between Treatment, Pupil Partner and either Sex Participant and/or Sex Partner that survived our threshold. In bold, effects *p* < .005.

**Table S6**

| **Fixed Factors** | | | | **F** | | **df1** | | **df2** | | ***p*-value** | |  |
| --- | --- | --- | --- | --- | --- | --- | --- | --- | --- | --- | --- | --- |
| Intercept | | | | 233.599 | | 1 | | 3575694373.211 | | **.000** | |  |
| Sex Participant | | | | .058 | | 1 | | 3576282248.333 | | .810 | |  |
| Treatment | | | | 3.046 | | 1 | | 226892.966 | | .081 | |  |
| Sex Partner | | | | .574 | | 1 | | 225749.821 | | .449 | |  |
| Pupil Partner | | | | .359 | | 1 | | 225747.157 | | .549 | |  |
| Group Partner | | | | 3.260 | | 1 | | 225747.593 | | .071 | |  |
| lin | | | | 204.862 | | 1 | | 874.287 | | **.000** | |  |
| Sex Participant * Treatment | | | | 3.958 | | 1 | | 226893.077 | | .047 | |  |
| Sex Participant * Sex Partner | | | | .278 | | 1 | | 225749.794 | | .598 | |  |
| Sex Participant * Pupil Partner | | | | .060 | | 1 | | 225747.077 | | .807 | |  |
| Sex Participant * Group Partner | | | | 1.034 | | 1 | | 225747.619 | | .309 | |  |
| Sex Participant * lin | | | | .705 | | 1 | | 806.232 | | .401 | |  |
| Treatment * Sex Partner | | | | .686 | | 1 | | 225748.637 | | .408 | |  |
| Treatment * Pupil Partner | | | | 6.921 | | 1 | | 225747.120 | | .009 | |  |
| Treatment * Group Partner | | | | 3.400 | | 1 | | 225746.176 | | .065 | |  |
| Treatment * lin | | | | 4.667 | | 1 | | 546.517 | | .031 | |  |
| Sex Partner * Pupil Partner | | | | 1.334 | | 1 | | 225749.363 | | .248 | |  |
| Sex Partner * Group Partner | | | | 1.264 | | 1 | | 22575.995 | | .261 | |  |
| Sex Partner * lin | | | | .028 | | 1 | | 225914.214 | | .866 | |  |
| Pupil Partner * Group Partner | | | | .770 | | 1 | | 225746.375 | | .380 | |  |
| Pupil Partner * lin | | | | .112 | | 1 | | 225889.289 | | .738 | |  |
| Group Partner * lin | | | | 23.939 | | 1 | | 225878.869 | | **.000** | |  |
| Sex Participant * Treatment * Sex Partner | | | | .074 | | 1 | | 225748.663 | | .786 | |  |
| Sex Participant * Treatment * Pupil Partner | | | | .712 | | 1 | | 225748.867 | | .399 | |  |
| Sex Participant * Treatment * Group Partner | | | | 2.874 | | 1 | | 225748.780 | | .090 | |  |
| Sex Participant * Treatment * lin | | | | 1.921 | | 1 | | 45.827 | | **.001** | |  |
| Sex Participant * Sex Partner * Pupil Partner | | | | 9.040 | | 1 | | 225748.689 | | **.003** | |  |
| Sex Participant * Sex Partner * Group Partner | | | | .045 | | 1 | | 225751.077 | | .831 | |  |
| Sex Participant * Sex Partner * lin | | | | .472 | | 1 | | 225933.844 | | .492 | |  |
| Sex Participant * Pupil Partner * Group Partner | | | | .026 | | 1 | | 225746.377 | | .873 | |  |
| Sex Participant * Pupil Partner * lin | | | | 5.893 | | 1 | | 225898.561 | | .015 | |  |
| Treatment * Pupil Partner * lin | | | | 15.975 | | 1 | | 22590.950 | | **.000** | |  |
| Treatment * Group Partner * lin | | | | 23.728 | | 1 | | 225889.604 | | **.000** | |  |
| Sex Partner * Group Partner * lin | | | | 13.063 | | 1 | | 225958.976 | | **.000** | |  |
| quadr | | | | 199.520 | | 1 | | 541.468 | | **.000** | |  |
| Sex Participant * quadr | | | | 5.813 | | 1 | | 541.471 | | .016 | |  |
| Treatment * quadr | | | | .024 | | 1 | | 339.290 | | .876 | |  |
| Sex Partner * quadr | | | | .092 | | 1 | | 226024.241 | | .762 | |  |
| Pupil Partner * quadr | | | | 15.381 | | 1 | | 225966.099 | | **.000** | |  |
| Group Partner * quadr | | | | 2.315 | | 1 | | 225945.336 | | .128 | |  |
| Sex Participant * Treatment * quadr | | | | .998 | | 1 | | 339.277 | | .318 | |  |
| Sex Participant * Sex Partner * quadr | | | | .631 | | 1 | | 226023.800 | | .427 | |  |
| Sex Participant * Pupil Partner * quadr | | | | 7.227 | | 1 | | 225962.219 | | .007 | |  |
| Sex Participant * Group Partner * quadr | | | | .199 | | 1 | | 225948.229 | | .655 | |  |
| Treatment * Sex Partner * Pupil Partner | | | | .469 | | 1 | | 225749.546 | | .493 | |  |
| Treatment * Sex Partner * Group Partner | | | | .448 | | 1 | | 225754.328 | | .503 | |  |
| Treatment * Sex Partner * quadr | | | | 6.330 | | 1 | | 226025.448 | | .012 | |  |
| Treatment * Pupil Partner * quadr | | | | 9.794 | | 1 | | 225963.865 | | **.002** | |  |
| Treatment * Group Partner * quadr | | | | 4.591 | | 1 | | 225951.275 | | .032 | |  |
| Sex Partner * Pupil Partner * Group Partner | | | | .808 | | 1 | | 22575.002 | | .369 | |  |
| Sex Partner * Pupil Partner * quadr | | | | 1.597 | | 1 | | 22597.273 | | .206 | |  |
| Sex Partner * Group Partner * quadr | | | | 6.979 | | 1 | | 226052.817 | | .008 | |  |
| Pupil Partner * Group Partner * quadr | | | | 3.803 | | 1 | | 225971.665 | | .051 | |  |
| Sex Participant * Treatment * Sex Partner * quadr | | | | 7.305 | | 1 | | 226025.484 | | .007 | |  |
| Sex Participant * Pupil Partner * Group Partner * quadr | | | | 8.981 | | 1 | | 225972.102 | | **.003** | |  |
| Sex Partner * Pupil Partner * Group Partner * quadr | | | | 6.089 | | 1 | | 226004.805 | | .014 | |  |
| cub | | | | 8.814 | | 1 | | 631.810 | | **.003** | |  |
| Treatment * cub | | | | 4.769 | | 1 | | 143393.796 | | .029 | |  |
| Sex Partner * cub | | | | 4.414 | | 1 | | 225917.414 | | .036 | |  |
| Pupil Partner * cub | | | | 5.307 | | 1 | | 225876.931 | | .021 | |  |
|  | | | |  | |  | |  | **95% Confidence Interval** | | |  |
| **Random Factors** | | **Estimate** | **SE** | | **Z** | | ***p*-value** | | **Lower** | | **Upper Bound** | |
| Repeated Measures | AR1 diagonal | .013 | .000 | | 335.963 | | **.000** | | .013 | | .013 | |
| lin [subject = ID] | Variance | .000 | .000 | | 7.549 | | **.000** | | .000 | | .001 | |
| quadr [subject = ID] | Variance | .000 | .000 | | 3.846 | | **.000** | | .000 | | .000 | |
| cub [subject = ID] | Variance | .000 | .000 | | 4.523 | | **.000** | | .000 | | .000 | |

**Pupil Constriction Mimicry- Pupil-Contingent Distrust Linkage**

There were no interactions between Treatment, Pupil Constriction Mimicry and either Sex Participant and/or Sex Partner that survived our threshold.

| **Table S7 Pupil Constriction Mimicry- Pupil-Contingent Distrust Linkage** | | | | | | |
| --- | --- | --- | --- | --- | --- | --- |
|  | | |  |  |  |  |
| **Fixed Factors** | | | **F** | **df1** | **df2** | ***p*-value** |
| Intercept | | | 75.048 | 1 | 96.744 | **.000** |
| Treatment | | | 1.037 | 1 | 96.744 | .311 |
| Constriction Mimicry | | | 5.451 | 1 | 1441.258 | .020 |
| Treatment * Constriction Mimicry | | | 7.053 | 1 | 1441.258 | .008 |
| Sex Partner | | | .155 | 1 | 1389.635 | .694 |
| Sex Participant | | | .009 | 1 | 96.744 | .924 |
| Treatment * Sex Partner | | | 2.297 | 1 | 1389.635 | .130 |
| Treatment * Sex Participant | | | 1.025 | 1 | 96.744 | .314 |
| Sex Partner * Sex Participant | | | 3.857 | 1 | 1389.635 | .050 |
| Sex Partner * Constriction Mimicry | | | 4.410 | 1 | 1453.363 | .036 |
| Sex Participant * Constriction Mimicry | | | .419 | 1 | 1441.258 | .518 |
| Treatment * Sex Partner * Sex Participant | | | .874 | 1 | 1389.635 | .350 |
| Treatment * Sex Partner * Constriction Mimicry | | | .006 | 1 | 1453.363 | .936 |
| Treatment * Sex Participant * Constriction Mimicry | | | 8.846 | 1 | 1441.258 | .003 |
| Sex Partner * Sex Participant * Constriction Mimicry | | | .004 | 1 | 1453.363 | .950 |
| Treatment * Sex Partner * Sex Participant * Constriction Mimicry | | | 5.526 | 1 | 1453.363 | .019 |
|  |  |  |  |  | **95% Confidence Interval** | |
| **Random Factors** | **Estimate** | **SE** | **Z** | ***p*-value** | **Lower** | **Upper Bound** |
| Residual | 3.500 | .133 | 26.312 | **.000** | 3.249 | 3.771 |
| Intercept [subject = ID * Session] Variance | .557 | .114 | 4.871 | **.000** | .373 | .834 |

**Looking Times on the Eye Region**

As can be seen in the table below, there was a three-way interaction between Treatment, Pupil Partner and Group Partner, supported by two two-way interactions between Pupil Partner and Group Partner and by the Treatment by Group Partner interaction. Follow-up tests revealed that participants under oxytocin versus placebo spend more time looking into the eyes of outgroup partners with dilating pupils *t*(128.256) = 2.340, *p* = .019.

| **Table S8. Looking Times on the Eye Region** | | | | |
| --- | --- | --- | --- | --- |
| **Fixed Factors*** | **F** | **df1** | **df2** | ***p*-value** |
| Corrected Model | 3,396 | 11 | 128.256 | **.000** |
| Treatment | 1,363 | 1 | 128.256 | .243 |
| Pupil Partner | 2,638 | 2 | 128.256 | .072 |
| Group Partner | 1,892 | 1 | 128.256 | .169 |
| Treatment * Pupil Partner | 0,094 | 2 | 128.256 | .911 |
| Treatment * Group Partner | 5,093 | 1 | 128.256 | .024 |
| Pupil Partner * Group Partner | 7,077 | 2 | 128.256 | **.001** |
| Treatment * Pupil Partner * Group Partner | 6,433 | 2 | 128.256 | .002 |
|  |  |  |  |  |
| **Random Factors** | **Estimate** | **SE** | **Z** | ***p*-value** |
| AR1 Diagonal ID * Session * Trial | .791 | .004 | 198.124 | **.000** |
| AR1 Rho ID * Session * Trial | .496 | .002 | .199.650 | **.000** |
| Var(Intercept) ID | 1.492 | .416 | 3.583 | **.000** |
| Var(Intercept) ID * Session | .648 | .181 | 3.581 | **.000** |

**Looking Times on the Eye Region**

We also investigated whether sex of the participant or the partner impacted on the looking times but none of the effects survived the threshold of *p* < .001 which we set for sex effects. Following up the interaction between Treatment, Pupil Partner and Group Partner, *t*-tests showed that participants, when treated with oxytocin as compared to placebo, attended longer to the eye region of outgroup partners with dilating pupils *t*(128.240) = 2.222, *p* = .026. A similar trend towards significance for treatment was found for ingroup partners with constricting pupils *t*(128.240) = 1.650, *p* = .099.

| **Table S9. Looking Times on the Eye Region** |  |  |  |  |
| --- | --- | --- | --- | --- |
| **Fixed factors** | **F** | **df1** | **df2** | ***p*-value** |
| Corrected Model | 3.396 | 27 | 128.240 | **.000** |
| Sex Participant | 2.426 | 1 | 128.240 | .119 |
| Treatment | .983 | 1 | 128.240 | .321 |
| Sex Partner | 3.615 | 1 | 128.240 | .057 |
| Pupil Partner | 2.128 | 2 | 128.240 | .119 |
| Group Partner | 2.207 | 1 | 128.240 | .137 |
| Sex Participant*Treatment | .404 | 1 | 128.240 | .525 |
| Sex Participant*Sex Partner | .031 | 1 | 128.240 | .861 |
| Sex Participant*Pupil Partner | 1.401 | 2 | 128.240 | .246 |
| Sex Participant* Group Partner | 2.45 | 1 | 128.240 | .118 |
| Treatment*Sex Partner | 8.496 | 1 | 128.240 | .004 |
| Treatment*Pupil Partner | .374 | 2 | 128.240 | .688 |
| Treatment* Group Partner | 4.578 | 1 | 128.240 | .032 |
| Sex Partner*Pupil Partner | .648 | 2 | 128.240 | .523 |
| Sex Partner* Group Partner | 4.26 | 1 | 128.240 | .039 |
| Pupil Partner* Group Partner | 6.481 | 2 | 128.240 | .002 |
| Sex Participant*Treatment*Sex Partner | 5.04 | 1 | 128.240 | .025 |
| Sex Participant*Treatment*Pupil Partner | 4.634 | 2 | 128.240 | .010 |
| Treatment*Pupil Partner*Group Partner | 7.496 | 2 | 128.240 | **.001** |
| Sex Partner*Pupil Partner*Group Partner | 4.939 | 2 | 128.240 | .007 |
|  |  |  |  |  |
| **Random Factors** | **Estimate** | **SE** | **Z** | ***p*-value** |
| AR1 Diagonal ID * Session * Trial | .808 | .004 | 199.445 | **.000** |
| Intercept [subject = ID * Session] Variance | .489 | .002 | 195.707 | **.000** |
| Var(Intercept) ID | 1.466 | .416 | 3.522 | **.000** |
| Var(Intercept) ID * Session | .657 | .185 | 3.547 | **.000** |

**Effects of baseline-pupil size, possibly indicative for the level of arousal or attention to the task**

In order to rule out that effects of sex of the participant or treatment on stimulus-induced pupil size were confounded by differences in pupil size that are not related to the stimulus, we also investigated pupil size during the presentation of the fixation cross.

| **Table S10. Pupil size during fixation cross** | | | | |
| --- | --- | --- | --- | --- |
| **Fixed Factors*** | **F** | **df1** | **df2** | ***p*-value** |
| Corrected Model | 1,168 | 3 | 25.487 | .320 |
| Treatment | 0,013 | 1 | 25.487 | .909 |
| Sex Participant | 0,288 | 1 | 25.487 | .592 |
| Sex Participant * Treatment | 3,148 | 1 | 25.487 | .076 |
|  |  |  |  |  |
| **Random Factors** | **Estimate** | **SE** | **Z** | ***p*-value** |
| AR1 Diagonal ID * Session * Trial | .031 | .001 | 50.083 | **.000** |
| AR1 Rho ID * Session * Trial | .994 | .000 | 7,890.922 | **.000** |
| Var(Intercept) ID | .114 | Redundant |  |  |
| Var(Intercept) ID * Session | .230 | Redundant |  |  |

**References**

Aron, A., Aron, E. N., & Smollan, D. (1992). Inclusion of other in the self scale and the structure of interpersonal closeness. *J Pers Soc Psychol, 63*(596-612).

Matsumoto, D., Ekman, P. (1989). American-Japanese cultural differences in judgments of facial expressions of emotion. *Motivation and Emotion, 13*, 143-157.

van der Schalk, J., Hawk, S. T., Fischer, A. H., & Doosje, B. (2011). Moving faces, looking places: validation of the Amsterdam Dynamic Facial Expression Set (ADFES). *Emotion, 11*(4), 907-920.
